# Open-label randomised pragmatic trial (CONTACT) comparing naproxen and low-dose colchicine for the treatment of gout flares in primary care

**DOI:** 10.1136/annrheumdis-2019-216154

**Published:** 2019-10-30

**Authors:** Edward Roddy, Kris Clarkson, Milica Blagojevic-Bucknall, Rajnikant Mehta, Raymond Oppong, Anthony Avery, Elaine M Hay, Carl Heneghan, Liz Hartshorne, Julie Hooper, Gemma Hughes, Sue Jowett, Martyn Lewis, Paul Little, Karen McCartney, Kamal R Mahtani, David Nunan, Miriam Santer, Sam Williams, Christian D Mallen

**Affiliations:** 1 Primary Care Centre Versus Arthritis; School of Primary, Community and Social Care, Keele University, Keele, UK; 2 Haywood Academic Rheumatology Centre, Midland Partnership NHS Foundation Trust, Stoke-on-Trent, UK; 3 Keele Clinical Trials Unit, Keele University, Keele, UK; 4 Birmingham Acute Care Research/Heart of England NHS Foundation Trust/Institute of Applied Health Research (BCTU), University of Birmingham, Birmingham, UK; 5 Health Economics, University of Birmingham, Birmingham, UK; 6 Division of Primary Care, University of Nottingham, Nottingham, UK; 7 Nuffield Department of Primary Care Health Sciences, University of Oxford, Oxford, UK; 8 Primary Care and Population Sciences, University of Southampton, Southampton, UK

**Keywords:** gout, primary care, naproxen, colchicine, randomised trial

## Abstract

**Objectives:**

To compare the effectiveness and safety of naproxen and low-dose colchicine for treating gout flares in primary care.

**Methods:**

This was a multicentre open-label randomised trial. Adults with a gout flare recruited from 100 general practices were randomised equally to naproxen 750 mg immediately then 250 mg every 8 hours for 7 days or low-dose colchicine 500 mcg three times per day for 4 days. The primary outcome was change in worst pain intensity in the last 24 hours (0–10 Numeric Rating Scale) from baseline measured daily over the first 7 days: mean change from baseline was compared between groups over days 1–7 by intention to treat.

**Results:**

Between 29 January 2014 and 31 December 2015, we recruited 399 participants (naproxen n=200, colchicine n=199), of whom 349 (87.5%) completed primary outcome data at day 7. There was no significant between-group difference in average pain-change scores over days 1–7 (colchicine vs naproxen: mean difference −0.18; 95% CI −0.53 to 0.17; p=0.32). During days 1–7, diarrhoea (45.9% vs 20.0%; OR 3.31; 2.01 to 5.44) and headache (20.5% vs 10.7%; 1.92; 1.03 to 3.55) were more common in the colchicine group than the naproxen group but constipation was less common (4.8% vs 19.3%; 0.24; 0.11 to 0.54).

**Conclusion:**

We found no difference in pain intensity over 7 days between people with a gout flare randomised to either naproxen or low-dose colchicine. Naproxen caused fewer side effects supporting naproxen as first-line treatment for gout flares in primary care in the absence of contraindications.

**Trial registration number:**

ISRCTN (69836939), clinicaltrials.gov (NCT01994226), EudraCT (2013-001354-95).

**Key messages**

**What is already known about this subject?**

- Non-steroidal anti-inflammatory drugs (NSAIDs) are effective treatments for gout flare, but side effects are frequent.
- Lower doses of colchicine are as effective as and better tolerated than high doses but have never been compared directly with an NSAID.

**What does this study add?**

- There was no difference between the effect of naproxen and low-dose colchicine on pain from gout flare.
- Naproxen was associated with fewer side effects, lower use of other analgesics and was cost-effective.

**How might this impact on clinical practice or future developments?**

- In the absence of contraindications, naproxen should be used ahead of low-dose colchicine in primary care on the grounds of effectiveness, safety and cost.

## Introduction

Gout affects 2.5% of adults in the UK and 3.8% in the USA.^1 2^ It causes sudden flares of excruciating joint pain and swelling, which are treated with non-steroidal anti-inflammatory drugs (NSAIDs), low-dose colchicine or corticosteroids.^3–5^

Numerous randomised trials demonstrate that NSAIDs treat gout flares effectively.^6 7^ However, side effects are frequent and can be life-threatening. NSAIDs are commonly used in all age groups: three-quarters of NSAID prescriptions for gout flares in the UK in 2001–2004 were for diclofenac or indomethacin,^8^ two of the most toxic NSAIDs.^9^ Naproxen is associated with lower vascular risk than other NSAIDs and is as effective as oral prednisolone for gout flares.^9 10^

High-dose colchicine is effective but commonly causes gastrointestinal side effects.^6 8 11–13^ Lower doses are as effective but better tolerated.^14^ The recommended ‘low-dose’ regimen in the UK is 500 mcg two to four times per day,^3 15^ however, the effectiveness and tolerability of this dose have never been evaluated. A direct comparison of an NSAID and low-dose colchicine is needed to inform choice for patients and practitioners.

The Colchicine Or Naproxen Treatment for ACute gouT (CONTACT) trial aimed to compare the clinical effectiveness of naproxen and low-dose colchicine at reducing pain from gout flares in primary care, their side-effect profiles and cost-effectiveness.

## Methods

### Study design

This was a randomised, multicentre, open-label, pragmatic clinical trial. The trial protocol is available at https://www.keele.ac.uk/pchs/research/inflammatoryconditions/contact/

### Participants

We recruited participants from 100 general practices across England. Registered patients who had consulted for gout in the preceding 2 years were mailed trial information before trial commencement and then 3 monthly inviting them to consult their general practitioner (GP) about the trial if they experienced a gout flare. Patients experiencing their first-ever flare were provided with trial information when they consulted.

Eligibility was assessed by the GP during a routine consultation. Participants were aged 18 years and over, consulting for a current gout flare, and had capacity and willingness to give consent and complete trial documentation. A clinical diagnosis of gout was made by the GP without joint aspiration, blood tests, imaging or diagnostic criteria. Exclusion criteria were unstable medical conditions (eg, ischaemic heart disease, impaired liver function); known stage 4/5 chronic kidney disease (estimated glomerular filtration rate/creatinine clearance <30 mL/min); recent surgery or gastrointestinal bleed; history of gastric ulcer; current anticoagulant use; allergy to aspirin or NSAID; previous inability to tolerate naproxen or low-dose colchicine; other contraindication to either study drug described in the Summary of Product Characteristics; prescription of naproxen or colchicine in the previous 24 hours; pregnancy or lactation; potentially vulnerable patients; and participation in the CONTACT trial during a previous gout flare or involvement in another clinical trial in the last 90 days or other research within the last 30 days. Written informed consent was obtained prior to participation.

### Randomisation, masking and interventions

Participants were randomly allocated 1:1 using simple randomisation to either:

1. Single initial dose of oral naproxen 750 mg (three 250 mg tablets) followed by 250 mg (one tablet) every 8 hours for up to 7 days. Co-prescription of a proton-pump inhibitor was at the GP’s discretion.
2. Oral colchicine 500 mcg (one tablet) every 8 hours for 4 days. Participants prescribed a statin were advised to omit the statin during colchicine treatment.

Randomisation was undertaken by the healthcare professional using web-access to a secure remote allocation system or, if this could not be accessed, a telephone randomisation service. Clinicians did not know which treatment a participant would receive prior to randomisation ensuring allocation concealment.

The GP prescribed the allocated medication. Participants and treating clinicians were aware of treatment allocation. Participants received a drug-specific advice leaflet that included advice about non-pharmacological treatment (rest, application of ice) and were offered reimbursement for prescription charges.

### Data collection

Baseline data were collected by self-complete questionnaire prior to randomisation. Outcome measures were collected by self-complete daily diary (days 1–7) and a questionnaire at week 4. On study entry, participants chose between paper (postal) or web-based (e-mail invitation) follow-up. Reminders were sent during week 1 (postcard or daily e-mail reminders). If diary data were not received by day 10, a blinded research nurse telephoned participants to capture key outcome data. Non-responders to the 4-week questionnaire were sent postal/e-mail reminders at 2 weeks and 4 weeks after initial mailing. Non-responders to the second reminder were telephoned by the research nurse and, if not successfully contacted, mailed a brief questionnaire.

Participants provided consent for review of their medical records over the 4-week study period to capture serious adverse events including hospitalisations and deaths.

### Outcomes

On days 0–7 and at week 4, participants rated the intensity of the worst pain experienced in the last 24 hours using a validated 0–10 Numeric Rating Scale (NRS).^16^ The primary outcome was change in pain intensity from baseline measured over the first 7 days. Secondary outcomes were time-to-treatment effect; complete pain resolution (reporting 0 or 1 on NRS); self-reported side effects (nausea, vomiting, headache, skin rash, dyspepsia, abdominal pain, constipation and diarrhoea); patient global assessment of treatment response (completely better/much better/somewhat better/about the same/somewhat worse/much worse); use of corticosteroids, paracetamol, NSAIDs or opiates for gout pain; treatment adherence; relapse/recurrent gout flare; quality of life (EQ-5D-5L)^17^; attendance at GP, emergency department or primary care out-of-hours service; and absence from work/education. Worst pain intensity in the last 24 hours, side effects, medication use for gout pain and treatment adherence were assessed daily during days 1–7 and at week 4. EQ-5D-5L and patient global assessment of treatment response were assessed at day 7 and week 4. Relapse/recurrent gout flare, re-attendance and work absence were assessed at week 4.

### Sample size

We aimed to assess the superiority of naproxen or colchicine (two-tailed hypothesis testing). A sample size of 200 participants per arm was required to detect a small standardised effect size (ES) of 0.3, allowing for the repeated measures structure (assumed autocorrelation 0.6), 20% loss to follow-up, 1:1 allocation ratio, 90% power and two-sided type 1 error of 0.05.^18^

### Statistical analysis

The main analysis was by intention-to-treat (ITT) evaluating participants as per allocation assignment. Mean change in worst pain intensity in the last 24 hours from baseline to each follow-up time point was calculated for each group. Analysis of the primary outcome was by linear mixed model with autoregressive covariance for repeated measures.^19^ Between-group mean differences for each day (and at week four) were derived from the group×time interaction within the model. Standardised between-group mean differences for pain were expressed as the estimated mean differences relative to the baseline SD of pain scores (ES).^18^ Analyses of primary and secondary outcomes were performed before and after adjustment for baseline pain score, age and gender.

The proportion of participants reporting complete pain resolution and time-to-first resolution of pain was compared between groups through χ^2^ and Mann-Whitney U tests, respectively. Stepped per-protocol evaluations of between-group difference in the primary outcome were undertaken by excluding: (1) protocol violators related to treatment and eligibility; (2) those who did not take their designated treatment at any point and (3) those who did not take the full treatment course (naproxen <7 days, colchicine <4 days).

Binary or ordinal logistic models were used to estimate ORs for between-group comparisons of secondary outcomes: patient global assessment of treatment response; relapse/recurrent gout flare; re-attendance; time off work because of gout; use of other medications for gout pain; and side effects, based on complete data and multiple imputation (MI) using chained equations based on 50 imputed data sets including treatment and sociodemographic variables as predictors. Separate MI evaluations were undertaken to maintain reasonable cases-to-variables ratio >5^20^: imputed variables comprised (i) primary/secondary health variables (excluding side effects) across baseline and follow-up and health utilisation at week 4 and (ii) key health variables (ie, pain, global response, EQ-5D-5L) plus days 1–7 and week 4 medication and side-effect variables.^21^ Number needed to treatment harm was estimated for side effects as the reciprocal of the absolute risk difference.^22^

In a sensitivity analysis, between-group differences in the primary outcome based on more inclusive baseline covariates including adjustment for first episode, age at first flare, location of gout, EQ-5D-5L, index of deprivation (fixed factors) and GP practice (random factor) were examined via MI evaluation (imputation data set (i) above).

Analysis was performed when all participants had completed follow-up; no interim analysis was performed. Primary and secondary outcomes (except per-protocol and health economic evaluations) were analysed blind to treatment allocation. The primary endpoint analysis was independently analysed by two statisticians. All analyses were carried out using SPSS V.21.0 and STATA V.14.0.

### Health economics

An incremental cost-utility analysis from a National Health Service (NHS)/personal social services perspective was undertaken. Unit costs (2015/2016 prices) from standard UK sources were applied to resource use data. EQ-5D-5L index scores were generated using the UK value set to calculate QALYs over the 4-week follow-up period.^23^

Resource use, costs and EQ-5D-5L scores were summarised using descriptive statistics. Missing EQ-5D-5L scores and costs were imputed using MI. QALYs were calculated for each participant using EQ-5D-5L responses. A regression approach controlled for imbalances in baseline EQ-5D-5L scores between treatment arms. Mean costs were estimated by treatment arm and the difference in mean costs (95% CI) calculated using non-parametric bootstrapping.^24^

Incremental cost-effectiveness ratios were estimated by dividing the mean cost difference between arms by the difference in mean QALYs. Five thousand pairs of mean cost and QALY differences were estimated by non-parametric bootstrapping and presented on a cost-effectiveness plane. Cost-effectiveness acceptability curves were plotted to determine the probability that naproxen was cost-effective.^25^

The human capital approach was used to estimate productivity costs from employment status and days off work due to health. The average wage for each respondent was identified using UK Standard Occupational Classification coding and annual earnings data.^26^

### Patient and public involvement

This trial was developed with research users with gout who provided feedback on the proposed recruitment and consent processes and choice of trial outcomes. Two patient representatives sat on the independent trial steering committee, playing a full part in monitoring trial progress and conduct, and provided advice on the design of questionnaires and Participant Information Leaflets.

## Results

**Figure 1:**
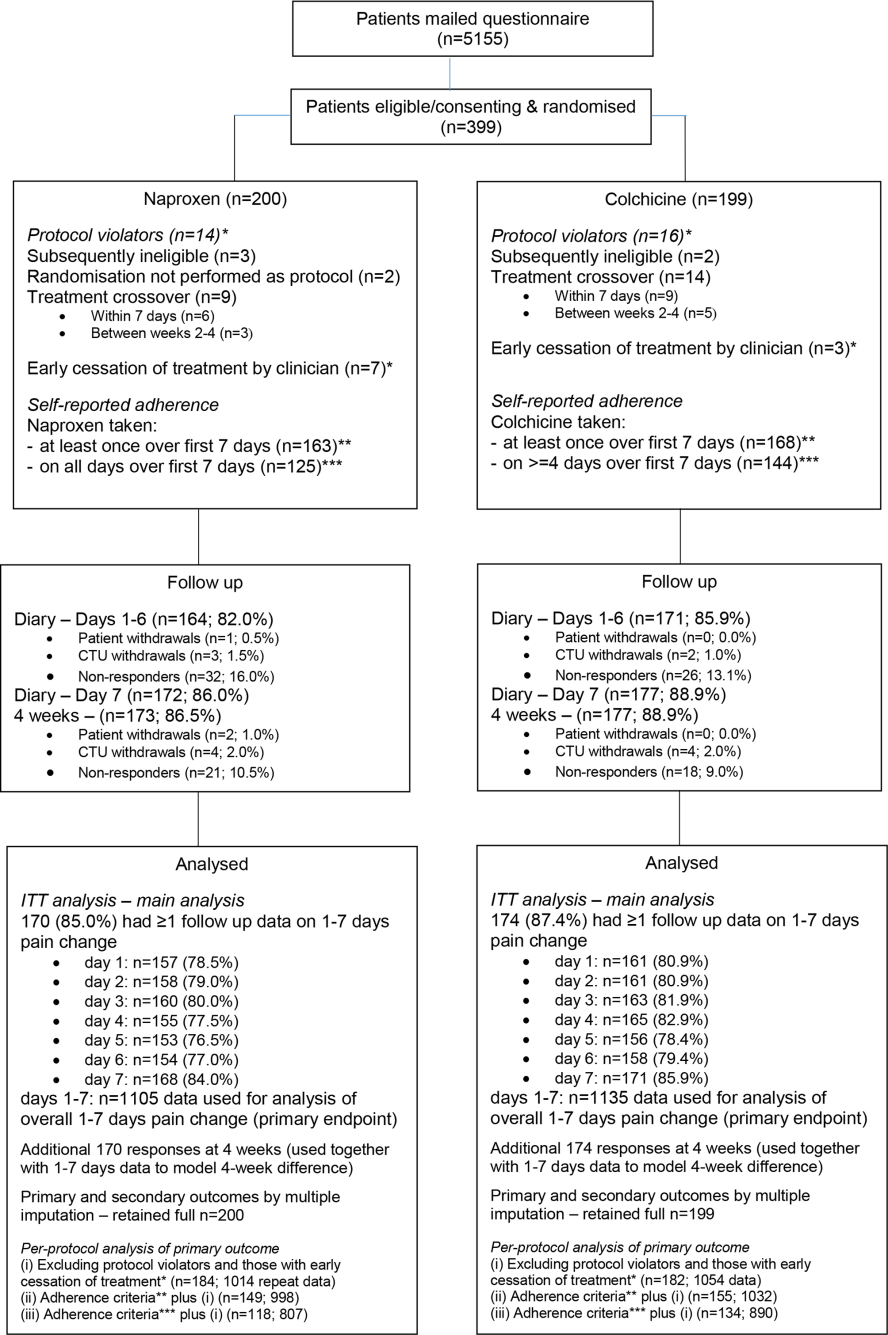
Participant flow. CTU, Clinical Trials Unit. ITT, intention-to-treat.

Between 29 January 2014 and 31 December 2015, 5155 patients were mailed. Three-hundred and ninety-nine participants were randomised: 200 to receive naproxen and 199 to receive colchicine (figure 1). Groups were similar at baseline although more people allocated to colchicine reported experiencing their first-ever gout flare (table 1, online supplementary table 1). Primary outcome data were collected for 86.0% in the naproxen group at day 7 and 86.5% at 4 weeks and 88.9% in the colchicine group at both day 7 and 4 weeks (figure 1).

10.1136/annrheumdis-2019-216154.supp1 Supplementary data

**Table 1:** Baseline characteristics

Key characteristics	Categories	Naproxen	Colchicine
Age: mean (SD)	–	58.7 (14.4)	60.0 (13.4)
Male, n (%)		173 (86.5)	174 (87.4)
Pain NRS (0–10), mean (SD)	–	7.1 (2.1)	6.9 (2.2)
	Missing data	7	5
First instance of gout, n (%)	–	35 (17.9)	51 (26.2)
	Missing data	4	4
Age when diagnosed, mean (SD)		52.1 (15.2)	53.4 (14.6)
	Missing data	6	7
Body part affected, n (%)	First MTPJ	142 (72.4)	135 (69.2)
	Other foot joints	58 (29.6)	48 (24.6)
	Other lower limb	46 (23.5)	47 (24.1)
	Upper limb	23 (11.7)	31 (15.9)
	Missing data	4	4
Number of body parts affected, n (%)	1	139 (70.9)	145 (74.3)
	2	34 (17.3)	27 (13.8)
	3	13 (6.6)	9 (4.6)
	4	6 (3.1)	13 (6.7)
	≥5	4 (2.0)	1 (0.5)
	Missing data	4	4
EQ-5D-5L, mean (SD)	–	0.665 (0.210)	0.666 (0.225)
	Missing data	8	6

MTPJ, metatarsophalangeal joint; NRS, Numeric Rating Scale.

There were 30 protocol violations (8% of participants) relating to treatment or eligibility (naproxen n=14, colchicine n=16). Of those returning diary data, 99% (163/164) reported taking the allocated treatment at least once and 75% (125) taking it on each day of the course in the naproxen group compared with 98% (168/171) and 85% (144), respectively, for colchicine.

**Figure 2:**
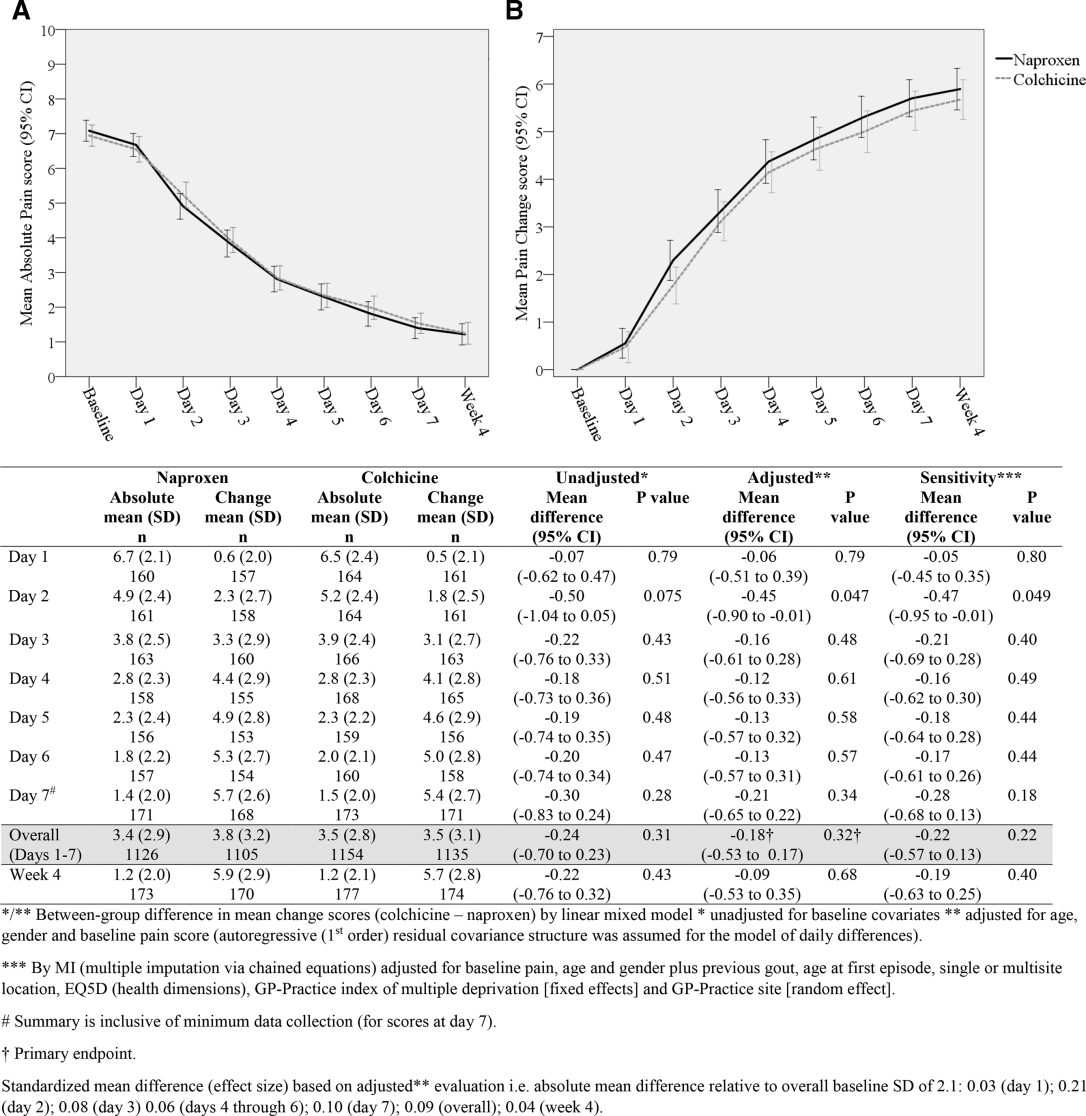
Comparison of pain scores (primary outcome measure) at follow-up (intention-to-treat analysis).

Within-group improvements in the primary outcome were seen in both groups over days 1–7 (figure 2). There was no significant between-group difference in mean change in worst pain intensity over days 1–7 (colchicine vs naproxen: adjusted mean difference −0.18; 95% CI −0.53 to 0.17; p=0.32; ES 0.09). Unadjusted estimates and MI evaluation with extended covariate adjustment were similar. There was a small between-group difference favouring naproxen on day 2 only.

Per-protocol analysis (1) showed comparable between-group mean differences to the ITT evaluation (online supplementary table 2). Per-protocol analyses (2) and (3) showed similar between-group differences to the ITT analysis overall and on days 1–6 but found small significant differences favouring naproxen at week 4.

There were no between-group differences in complete pain resolution or patient global assessment of treatment response at any time-point (table 2, online supplementary table 3). At week 4, there were no between-group differences in proportions reporting a relapse/recurrent gout flare; consulting a GP, practice nurse or emergency department; or time off work.

**Table 2:** Comparison of secondary outcome measures at day 7 and week 4 follow-up

	Naproxen	Colchicine	OR (95% CI) (p value)*	OR (95% CI) (p value)†
Complete pain resolution, n (%)				
7 days	115 (67.3)	116 (67.1)	0.96 (0.60 to 1.54) (p=0.87)	0.95 (0.60 to 1.48) (p=0.81)
4 weeks	130 (75.1)	130 (73.4)	0.83 (0.51 to 1.36) (p=0.46)	0.90 (0.56 to 1.44) (p=0.66)
Days to complete pain resolution, median (IQR)	5 (day 4, week 4)	6 (day 4, week 4)	–	–
Patient assessment of global treatment response (completely/much better), n (%)				
7 days	114 (71.3)	110 (72.4)	1.11 (0.67 to 1.84) (p=0.69)	1.03 (0.62 to 1.70) (p=0.91)
4 weeks	140 (80.9)	143 (80.8)	0.88 (0.51 to 1.52) (p=0.64)	0.96 (0.56 to 1.64)(p=0.87)
Recurrence/relapse of gout flare during 4-week follow-up, n (%)	40 (30.1)	54 (35.1)	1.28 (0.78 to 2.13) (p=0.33)	1.24 (0.77 to 1.99) (p=0.37)
Consultation/re-attendance for gout during 4-week follow-up, n (%)				
Health professional‡	30 (22.6)	41 (26.6)	1.43 (0.82 to 2.51) (p=0.213)	1.39 (0.82 to 2.34) (p=0.22)
GP	26 (19.4)	39 (25.3)	1.69 (0.93 to 3.05) (p=0.083)	1.56 (0.89 to 2.72) (p=0.12)
Number of times				
1	14 (58.3)	27 (69.2)	–	–
2	8 (33.3)	10 (25.6)		
3	2 (8.3)	2 (5.1)		
Practice nurse	7 (5.3)	10 (6.6)	1.31 (0.47 to 3.64) (p=0.61)	1.23 (0.45 to 3.32) (p=0.69)
Number of times				
1	5 (71.4)	9 (90.0)	–	–
2	1 (14.3)	1 (10.0)		
3	1 (14.3)	0 (0.0)		
Emergency GP	6 (4.5)	6 (3.9)	0.84 (0.26 to 2.68) (p=0.77)	0.87 (0.30 to 2.57) (p=0.81)
Emergency department	1 (0.8)	1 (0.7)	–	1.23 (0.07 to 21.5) (p=0.89)
Taken time off work because of gout during 4-week follow-up, n (%)	11 (8.6)	8 (5.3)	0.61 (0.22 to 1.64) (p=0.33)	0.76 (0.31 to 1.91) (p=0.57)
Days, median (IQR)	4 (2, 12)	3 (3, 17)	–	–

OR for colchicine relative to naproxen.

*Analysis of complete case data (adjusted for baseline pain, age and gender).

†Analysis through multiple imputation via chained equations with logistic (binary/ordinal) regression model (adjusted for age, sex and baseline pain) based on full ITT on 50 imputations.

‡Health professional: GP, practice nurse, emergency GP and/or accident and emergency.

GP, general practitioner; ITT, intention-to-treat.

More participants in the colchicine group used paracetamol or codeine for gout during days 1–7 than in the naproxen group (table 3). At week 4, ibuprofen use was more common in the colchicine group on complete case analysis but not in the MI data set.

**Table 3:** Use of medication for relief of gout pain over the first week (diary days 1–7) and between weeks 2 and 4 (week 4 follow-up)

	Days 1–7				Weeks 2–4			
	Naproxen	Colchicine	OR (95% CI) (p value)		Naproxen	Colchicine	OR (95% CI) (p value)	
	N (%)*	N (%)*	Complete case*	Imputed†	N (%)*	N (%)*	Complete case*	Imputed†
Paracetamol	20 (13.4)	34 (23.6)	2.09 (1.11 to 3.93) (p=0.022)	1.91 (1.05 to 3.51) (p=0.035)	10 (7.5)	11 (7.1)	1.12 (0.45 to 2.82) (p=0.81)	0.98 (0.40 to 2.37) (p=0.96)
Ibuprofen	16 (10.7)	20 (13.9)	1.54 (0.72 to 3.29) (p=0.27)	1.58 (0.80 to 3.12) (p=0.19)	12 (9.0)	27 (17.5)	2.34 (1.11 to 4.94) (p=0.026)	1.93 (0.90 to 4.14) (p=0.089)
Diclofenac	2 (1.3)	4 (2.8)	–	–	4 (3.0)	6 (3.9)	–	–
Indomethacin	1 (0.7)	0 (0.0)	–	–	2 (1.5)	5 (3.2)	–	–
Tramadol	1 (0.7)	0 (0.0)	–	–	1 (0.7)	2 (1.3)	–	–
Codeine	7 (4.7)	21 (14.6)	3.62 (1.47 to 8.93) (p=0.005)	3.20 (1.35 to 7.57) (p=0.008)	12 (9.0)	8 (5.2)	0.60 (0.22 to 1.65) (p=0.32)	0.59 (0.23 to 1.50) (p=0.27)
Prednisolone	3 (2.0)	2 (1.4)	–	–	2 (1.5)	1 (0.6)	–	–
Any analgesic or non-naproxen NSAID‡	37 (24.8)	61 (42.4)	2.23 (1.35 to 3.66) (p=0.001)	1.89 (1.24 to 2.88) (p=0.003)	37 (27.6)	52 (33.8)	1.34 (0.81 to 2.21) (p=0.26)	0.95 (0.63 to 1.43) (p=0.81)

OR for colchicine relative to naproxen (adjusted for age, gender and baseline pain score). *Analysis of complete case data (days 1–7: n=288; five cases excluded due to missing baseline pain scores; week 4: n=283; five cases excluded due to missing baseline pain scores). †Analysis of imputed data (n=399). n/a: analysis not applicable (as it is an evaluation of compliance with allocated treatment). –, ORs not estimated due to small frequency counts.

*Complete response to medication questions: diary days 1–7—149 in naproxen group and 144 in colchicine group; week 4—134 in naproxen group and 154 in colchicine group.

†Imputed data set: 200 in naproxen group; 199 in colchicine group (full ITT analysis).

‡Paracetamol or codeine or tramadol or ibuprofen or diclofenac or indomethacin.

ITT, intention-to-treat; NSAID, non-steroidal anti-inflammatory drug.

There were three serious adverse events, none related to trial interventions, and no deaths. Two participants who received naproxen were hospitalised: one for non-cardiac chest pain and one for hospital-acquired pneumonia following a transcatheter aortic valve implantation. One participant who received colchicine was hospitalised with osteomyelitis. During days 1–7, self-reported diarrhoea and headache were more common with colchicine than naproxen, whereas constipation was less common with colchicine (table 4). Diarrhoea peaked on day 4 in the colchicine group and constipation on day 3 in the naproxen group (online supplementary table 4). Both reduced considerably during weeks 2–4.

**Table 4:** Self-reported side effects over the first week and between weeks 2 and 4 (week 4 follow-up)

	Days 1–7					Weeks 2–4				
	Naproxen	Colchicine	OR (95% CI) {p value}		NNTH (95%CI)*	Naproxen	Colchicine	OR (95% CI) {p value}		NNTH (95%CI)*
	N (%)†	N (%)†	Complete case†	Imputed‡		N (%)†	N (%)†	Complete case†	Imputed‡	
Nausea and/or vomiting	21 (14.0)	30 (20.5)	1.82 (0.96 to 3.46) {p=0.066}	1.28 (0.71 to 2.30) {p=0.42}	31^1^ (8-∞^1^ to 27-∞^2^)	7 (5.2)	5 (3.2)	0.51 (0.14 to 1.83) {p=0.30}	0.59 (0.19 to 1.90) {p=0.38}	48^2^ (24-∞^2^ to 24-∞^1^)
Dyspepsia	20 (13.3)	20 (13.7)	0.89 (0.48 to 1.90) {p=0.95}	1.09 (0.58 to 2.04) {p=0.79}	98^1^ (9-∞^1^ to 19-∞^2^)	13 (9.7)	8 (5.2)	0.44 (0.17 to 1.15) {p=0.094}	0.59 (0.24 to 1.45) {p=0.25}	27^2^ (14-∞^2^ to 26-∞^1^)
Abdominal pain	16 (10.7)	16 (11.0)	1.07 (0.51 to 2.25) {p=0.86}	0.83 (0.40 to 1.71) {p=0.61}	53^2^ (16-∞^2^ to 16-∞^1^)	4 (3.0)	8 (5.2)	1.57 (0.44 to 5.53) {p=0.49}	1.32 (0.43 to 4.09) {p=0.63}	108^1^ (12-∞^1^ to 59-∞^2^)
Headache	16 (10.7)	30 (20.5)	2.38 (1.21 to 4.68) {p=0.012}	1.92 (1.03 to 3.55) {p=0.039}	12^1^ (5^1^ to 350^1^)	4 (3.0)	4 (2.6)	0.92 (0.22 to 3.86) {p=0.91}	0.80 (0.21 to 3.10) {p=0.75}	171^2^ (42-∞^2^ to 17-∞^1^)
Constipation	29 (19.3)	7 (4.8)	0.20 (0.08 to 0.48) {p<0.001}	0.24 (0.11 to 0.54) {p<0.001}	7^2^ (6^2^ to 13^2^)	9 (6.7)	6 (3.9)	0.49 (0.16 to 1.54) {p=0.22}	0.57 (0.21 to 1.55) {p=0.27}	36^2^ (19-∞^2^ to 31-∞^1^)
Diarrhoea	30 (20.0)	67 (45.9)	3.54 (2.10 to 5.99) {p<0.001}	3.31 (2.01 to 5.44) {p<0.001}	4^1^ (3^1^ to 7^1^)	5 (3.7)	10 (6.5)	1.75 (0.58 to 5.26) {p=0.32}	1.59 (0.54 to 4.66) {p=0.40}	49^1^ (9-∞^1^ to 60-∞^2^)
Skin rash	3 (2.0)	3 (2.1)	1.13 (0.22 to 5.83) {p=0.88}	1.06 (0.21 to 5.39) {p=0.95}	851^1^ (13-∞^1^ to 64-∞^2^)	3 (2.2)	3 (1.9)	0.98 (0.19 to 5.09) {p=0.98}	0.97 (0.19 to 5.03) {p=0.97}	1548^2^ (56-∞^2^ to 13-∞^1^)
Any side effect(s)§	91 (60.7)	101 (69.2)	1.49¶ (0.92 to 2.43) {p=0.11}¶	1.60 (1.03 to 2.49) {p=0.038}	10^1^ (5^1^ to 142^1^)	37 (27.6)	28 (18.2)	0.58 (0.33 to 1.03) {p=0.064}	0.71 (0.41 to 1.23) {p=0.23}	16^2^ (7-∞^2^ to 23-∞^1^)

OR for colchicine relative to naproxen (adjusted for age, gender and baseline pain score). †Analysis of complete data (days 1–7: n=291; five cases excluded due to missing baseline pain scores; week 4: n=283; five cases excluded due to missing baseline pain scores). ‡ Analysis of imputed data (n=399).

*Number needed to treatment harm (NNTH): ^1^for colchicine over naproxen; ^2^for naproxen over colchicine (based on imputed OR estimates and observed rates for the naproxen group).

†Complete response to side-effect questions: diary days 1–7 naproxen=150, colchicine=146; week 4 naproxen n=134, colchicine=154.

‡Imputed data set: 200 in naproxen group; 199 in colchicine group (full ITT analysis).^1^

§Includes the side effects listed and ‘other’ (nominated free-text) side effects.

ITT, intention-to-treat.

Naproxen was slightly less costly and more effective than colchicine (online supplementary table 5). At a willingness-to-pay threshold of £20 000 per QALY, naproxen had an 80% chance of being cost-effective compared with colchicine (online supplementary figure).

10.1136/annrheumdis-2019-216154.supp2 Supplementary data

## Discussion

We found substantial within-group improvements in pain intensity in both groups but no statistically significant difference between naproxen and low-dose colchicine over the first 7 days. Naproxen appeared to provide faster pain relief, which could be explained by the 750 mg loading dose although the between-group difference at day 2 was small and possibly spurious. Side effects, particularly diarrhoea, and analgesic use were more frequent with colchicine. There were no major harms with naproxen. Naproxen was slightly more cost-effective than colchicine. These findings suggest that naproxen should be considered ahead of low-dose colchicine to treat gout flares in primary care in the absence of contraindications.

This is the first head-to-head comparison of naproxen and colchicine for gout flares and the first randomised trial of colchicine at this dose. In an equivalence trial comparing naproxen and prednisolone for gout flare,^10^ mean pain reduction (0–100 mm visual analogue scale) was 46 mm with naproxen by day 4 similar to the 4.1 mean reduction in our trial. A reduction of 2 points on a 0–10 pain NRS has been shown to be clinically significant in chronic pain.^27^ Only 70% of participants were completely/much better by day 7 and 80% by week 4, consistent with clinical observations that flares often persist beyond 1 week and one-third of participants reporting a recurrent flare by week 4. There have been two placebo-controlled trials of colchicine for gout flare, one used a traditional high-dose regime^13^ whereas the AGREE trial included both high-dose and low-dose arms.^14^ Lower doses are recommended to lessen gastrointestinal side effects while maintaining effectiveness.^3–5^ We used the UK recommended dose of colchicine, which is intermediate to the regime used by Ahern *et al* and the AGREE trial.^13–15^ Forty-two per cent of participants reported diarrhoea in week 1 compared with 100% with the regime of Ahern *et al* and 77% and 23% in the AGREE trial high-dose and low-dose regimes, respectively. Eighteen per cent in the naproxen group reported diarrhoea, similar to 14% in the placebo group in the AGREE trial. It was unexpected that headache differed between the groups, but it is plausible that naproxen may have a protective effect to treat or prevent headaches. Colchicine is considered to be more effective if given in the first 12–36 hours of a flare.^4 5 14^ Two-thirds of our participants initiated medication over 24 hours after symptom-onset providing ‘real-world’ evidence that low-dose colchicine is effective even when treatment is delayed due to patient or service-related factors.

Strengths of this trial include its primary care setting and pragmatic design. Although this should ensure generalisability to most patients with gout who are managed in the community, we did not assess existing comorbidities, use of urate-lowering therapy or prior flare rates to verify this. Gout diagnosis was made clinically rather than using validated criteria or additional investigations risking misclassification, although clinical diagnosis of gout in UK primary care has a positive predictive value of 90%.^28^ Further limitations include the open-label design without blinded outcome assessment or placebo tablets, and collection of solely self-reported outcomes without assessing the effect of NSAIDs on objective measures such as blood pressure or renal function. More participants in the naproxen group had experienced gout in the past and hence probably taken trial medications previously, possibly influencing perception of treatment effect, although participating clinical staff were trained to maintain equipoise. Since having recurrent flares increases the likelihood of a correct diagnosis,^29^ misclassification could have been greater in the colchicine group. Hence, it is possible that the naproxen group could have been advantaged, if previous treatment experiences influenced outcome reporting or alternative diagnoses such as osteoarthritis or palindromic rheumatism respond better to NSAID than colchicine. Finally, recruitment fell one short of the target of 400 participants. However, follow-up was better than anticipated and exceeded the required number of participants at the primary end-point.

We chose the dose of naproxen specified for gout flares in its marketing authorisation,^30^ although two times per day dosing is not uncommon in clinical practice. A previous randomised trial demonstrated equivalence of naproxen 500 mg two times per day to prednisolone for gout flares.^10^ Colchicine treatment was limited to 4 days, consistent with UK guidance, which advises a maximum total dose of 6 mg per course.^15^ In contrast, the AGREE trial low-dose arm comprised a total dose of 1.8 mg over 2 hours,^14^ although the American College of Rheumatology gout guideline recommended that this can be followed by 600 mcg one time or two times per day until flare resolution.^4^ While the longer treatment duration could have biased towards naproxen, colchicine was effective within the treatment period and there were no statistically significant between-group differences between days 3 and 7.

NSAIDs and colchicine are not the only drugs used to treat gout flares. The American College of Physicians recommends corticosteroids as first-line treatment, whereas other guidelines advise being guided by comorbidities, contraindications, previous response and the pattern of joint involvement.^3–5 31^ While randomised trials have compared NSAIDs and prednisolone,^10^ future research should compare the effectiveness and safety of colchicine and corticosteroids, particularly in patients with contraindications to NSAIDs. We found little difference in pain reduction between naproxen and low-dose colchicine, but naproxen was associated with fewer side effects, less analgesic use and slightly lower costs, suggesting that, in the absence of contraindications, naproxen should be used ahead of low-dose colchicine to treat gout flares in primary care.
