# Supplementary material for: Open-label randomised pragmatic trial (CONTACT) comparing naproxen and low-dose colchicine for the treatment of gout flares in primary care
Source: Ann Rheum Dis. 2019 Oct 30;79(2):276–84. doi: 10.1136/annrheumdis-2019-216154 (PMC7025732; doi:10.1136/annrheumdis-2019-216154)
Supplement: Supplementary data [file annrheumdis-2019-216154supp001.pdf]

Supplementary Table 1: recruitment by region and GP practice

	Naproxen	Colchicine
**Recruitment by region, n (%)**		
Keele	55 (27.5%)	52 (26.1%)
Southampton	88 (44.0%)	101 (50.8%)
Oxford	41 (20.5%)	31 (15.6%)
Nottingham	16 (8.0%)	15 (7.5%)
**Participants recruited per practice, median (IQR)***	2 (1-4)	2 (1-3)

*75/100 GP practices who agreed to take part recruited at least one participant. Numbers recruited per practice ranged from 1 (21 practices) to 41 for the best recruiting practice (skewed upwards); overall median of 3 (IQR 1-7).
IQR, interquartile range

Roddy E, *et al. Ann Rheum Dis* 2020; 79:276–284. doi: 10.1136/annrheumdis-2019-216154

Supplementary table 2: per protocol analyses

	1		2		3	
	**Mean difference (95% CI)**	**P value**	**Mean difference (95% CI)**	**P value**	**Mean difference (95% CI)**	**P value**
Day 1	0.01 (-0.45 to 0.46)	0.97	-0.04 (-0.50 to 0.41)	0.85	-0.04 (-0.54 to 0.46)	0.88
Day 2	-0.43 (-0.88 to 0.02)	0.063	-0.47 (-0.92 to -0.02)	0.040	-0.57 (-1.07 to -0.07)	0.024
Day 3	-0.11 (-0.56 to 0.33)	0.62	-0.17 (-0.62 to 0.28)	0.47	-0.20 (-0.69 to 0.30)	0.44
Day 4	-0.11 (-0.56 to 0.34)	0.62	-0.19 (-0.64 to 0.26)	0.41	-0.23 (-0.72 to 0.27)	0.37
Day 5	-0.16 (-0.61 to 0.28)	0.47	-0.25 (-0.71 to 0.20)	0.27	-0.23 (-0.73 to 0.27)	0.36
Day 6	-0.18 (-0.63 to 0.26)	0.42	-0.31 (-0.77 to 0.14)	0.17	-0.22 (-0.72 to 0.27)	0.38
Day 7[#]	-0.28 (-0.72 to 0.16)	0.22	-0.43 (-0.88 to 0.02)	0.061	-0.41 (-0.91 to 0.08)	0.10
Overall (Days 1-7)	-0.18 (-0.54 to 0.17)	0.32	-0.27 (-0.62 to 0.09)	0.14	-0.27 (-0.67 to 0.13)	0.18
Week 4	-0.24 (-0.68 to 0.20)	0.29	-0.48 (-0.94 to -0.03)	0.038	-0.57 (-1.07 to -0.07)	0.025

Definition of per-protocol analyses:-

1 = No treatment violation by medical record note reporting (no treatment crossover + no early cessation of treatment) {n (A/B) = 184/182; 1014 / 1054 repeat data across days 1-7}

2 = At least 1-day use of randomised treatment by self-report (in addition to 1 above) {n (A/B) = 149/155; 998 / 1032 repeat data across days 1-7}

3 = 7 days (4-7 days) use of randomised treatment naproxen (colchicine) by self-report (in addition to 1 above) {n (A/B) = 118/134; 807 / 890 repeat data across days 1-7}

Summary is inclusive of minimum data collection (for scores at day 7)

Roddy E, *et al. Ann Rheum Dis* 2020; 79:276–284. doi: 10.1136/annrheumdis-2019-216154

Supplementary table 3: Comparison of complete pain resolution over days 1-7 and at 4 weeks

	Naproxen	Colchicine	$\chi 2$	P value
Day 1	2/160 (1.3%)	8/164 (4.9%)	3.6	0.10*
Day 2	11/161 (6.8%)	12/164 (7.3%)	0.03	0.87
Day 3	29/163 (17.8%)	29/166 (17.5%)	0.01	0.94
Day 4	56/158 (35.4%)	55/168 (32.7%)	0.27	0.61
Day 5	79/156 (50.6%)	72/159 (45.3%)	0.91	0.34
Day 6	95/157 (60.5%)	89/160 (55.6%)	0.78	0.38
Day 7	115/171 (67.3%)	116/173 (67.1%)	<0.01	0.97
Week 4	130/173 (75.1%)	130/177 (73.4%)	0.13	0.72

* Complete-case responders to all pain questions (diary days 1 through 7 and at 4 weeks).
Median = Day 5 for Naproxen; Median = Day 6 for Colchicine.
Interquartile range: Day 4, Week 4 [in both treatment groups].
P value by Mann-Whitney U test = 0.73.

Roddy E, *et al. Ann Rheum Dis* 2020; 79:276–284. doi: 10.1136/annrheumdis-2019-216154

Supplementary Table 4: Self-reported daily side-effects within the first week of follow-up – complete-case data

	**Day1**		**Day2**		**Day3**		**Day4**		**Day5**		**Day6**		**Day7**	
	N (n=160)	C (n=164)	N (n=161)	C (n=164)	N (n=163)	C (n=166)	N (n=160)	C (n=168)	N (n=160)	C (n=162)	N (n=158)	C (n=162)	N (n=172)*	C (n=176)*
	NP	CC	NP	CC	NP	CC	NP	CC	NP	CC	NP	CC	NP	CC
Nausea	17 (10.6%)	15 (9.1%)	11 (6.8%)	16 (9.8%)	7 (4.3%)	17 (10.2%)	6 (3.8%)	11 (6.5%)	6 (3.8%)	6 (3.7%)	2 (1.3%)	6 (3.7%)	3 (1.7%)	5 (2.8%)
Vomiting	3 (1.9%)	1 (0.6%)	2 (1.2%)	1 (0.6%)	1 (0.6%)	1 (0.6%)	0 (0.0%)	0 (0.0%)	0 (0.0%)	0 (0.0%)	0 (0.0%)	1 (0.6%)	0 (0.0%)	0 (0.0%)
Nausea and/or vomiting	19 (11.9%)	16 (9.8%)	12 (7.5%)	17 (10.4%)	7 (4.3%)	18 (10.8%)	6 (3.8%)	11 (6.5%)	6 (3.8%)	6 (3.7%)	2 (1.3%)	7 (4.3%)	3 (1.7%)	5 (2.8%)
Dyspepsia	10 (6.3%)	13 (7.9%)	11 (6.8%)	11 (6.7%)	11 (6.7%)	14 (8.4%)	9 (5.6%)	11 (6.5%)	5 (3.1%)	7 (4.3%)	6 (3.8%)	6 (3.7%)	4 (2.3%)	6 (3.4%)
Abdominal pain	5 (3.1%)	7 (4.3%)	6 (3.7%)	8 (4.9%)	7 (4.3%)	8 (4.8%)	7 (4.4%)	8 (4.8%)	3 (1.9%)	9 (5.6%)	2 (1.3%)	6 (3.7%)	4 (2.3%)	6 (3.4%)
Headache	9 (5.6%)	16 (9.8%)	10 (6.2%)	12 (7.3%)	3 (1.8%)	13 (7.8%)	3 (1.9%)	13 (7.7%)	5 (3.1%)	10 (6.2%)	4 (2.5%)	5 (3.1%)	3 (1.7%)	4 (2.3%)
Constipation	8 (5.0%)	1 (0.6%)	15 (9.3%)	2 (1.2%)	16 (9.8%)	5 (3.0%)	8 (5.0%)	4 (2.4%)	6 (3.8%)	3 (1.9%)	6 (3.8%)	2 (1.2%)	5 (2.9%)	3 (1.7%)
Diarrhoea	7 (4.4%)	20 (12.2%)	7 (4.3%)	28 (17.1%)	12 (7.4%)	40 (24.1%)	10 (6.3%)	52 (31.0%)	3 (1.9%)	34 (21.0%)	7 (4.4%)	18 (11.1%)	5 (2.9%)	13 (7.4%)
Skin rash	2 (1.3%)	3 (1.8%)	2 (1.2%)	2 (1.2%)	0 (0.0%)	1 (0.6%)	0 (0.0%)	1 (0.6%)	1 (0.6%)	1 (0.6%)	0 (0.0%)	1 (0.6%)	0 (0.0%)	2 (1.1%)
Any side effect(s)[#]	54 (33.8%)	54 (32.9%)	50 (31.1%)	64 (39.0%)	52 (31.9%)	76 (45.8%)	45 (28.1%)	78 (46.4%)	38 (23.8%)	52 (32.1%)	35 (22.2%)	36 (22.2%)	26 (15.1%)	32 (18.2%)

N = Naproxen; C = Colchicine

* These are higher denominator numbers since questions on side-effects on day 7 were included in minimal data collection retrieval.

[#] Includes the side-effects listed and 'other' (nominated free-text) side-effects.

Supplementary table 5: Mean (SD) resource use, costs and outcomes per participant over 4 weeks follow-up

	Naproxen (n=200)	Colchicine (n=199)	Difference (CI)
**RESOURCE USE**			
GP visits	0.19 (0.34)	0.27 (0.40)	-0.08 (-0.15 to 0.0002)
Nurse visits	0.05 (0.19)	0.07 (0.22)	-0.02 (-0.06 to 0.03)
Emergency GP visits	0.05 (0.17)	0.04 (0.18)	0.01 (-0.03 to 0.04)
A and E visits	0.006 (0.07)	0.007 (0.07)	-0.001 (-0.02 to 0.01)
**COSTS (£)**			
Drug costs	0.83 (2.00)	1.20 (2.22)	-0.37 (-0.78 to 0.02)
GP costs	6.44 (11.16)	8.80 (13.16)	-2.36 (-4.74 to 0.12)
Nurse costs	0.66 (2.26)	0.86 (2.71)	-0.20 (-0.68 to 0.31)
Emergency GP costs	2.45 (8.54)	2.14 (8.68)	0.31 (-1.28 to 2.04)
A and E costs	0.41 (5.10)	0.48 (5.15)	-0.07 (-1.21 to 0.95)
Intervention cost	6.77 (4.56)	9.83 (6.32)	-3.06 (-4.17 to -2.08)
Total cost	17.57 (20.38)	23.31 (23.46)	-5.74 (-10.03 to -1.64)
**HEALTH OUTCOMES**			
Baseline EQ-5D	0.665 (0.21)	0.663 (0.22)	0.002 (-0.04 to 0.04)
Day 7 EQ-5D	0.882 (0.13)	0.873 (0.14)	0.009 (-0.02 to 0.03)
Week 4 EQ-5D	0.900 (0.11)	0.894 (0.15)	0.006 (-0.02 to 0.03)
QALYs	0.0663 (0.008)	0.0657 (0.01)	0.0006 (-0.001 to 0.002)
Adjusted QALYs$^+$	0.0662	0.0658	0.0004
**WORK-RELATED OUTCOMES**			
Time off work (days)	0.40 (2.47)	0.35 (2.51)	0.05 (-0.42 to 0.53)
Productivity cost (£)	32.16 (190.40)	28.44(207.42)	3.72 (-34.27 to 40.73)

$^+$*Adjusted for baseline EQ-5D*

Roddy E, *et al. Ann Rheum Dis* 2020; 79:276–284. doi: 10.1136/annrheumdis-2019-216154
