# Supplementary material for: Open-label randomised pragmatic trial (CONTACT) comparing naproxen and low-dose colchicine for the treatment of gout flares in primary care
Source: Ann Rheum Dis. 2019 Oct 30;79(2):276–84. doi: 10.1136/annrheumdis-2019-216154 (PMC7025732; doi:10.1136/annrheumdis-2019-216154)

Supplementary figure: Cost-effectiveness plane and acceptability curve for the comparison of Naproxen vs Colchicine

(A) Cost-effectiveness plane for Naproxen vs Colchicine

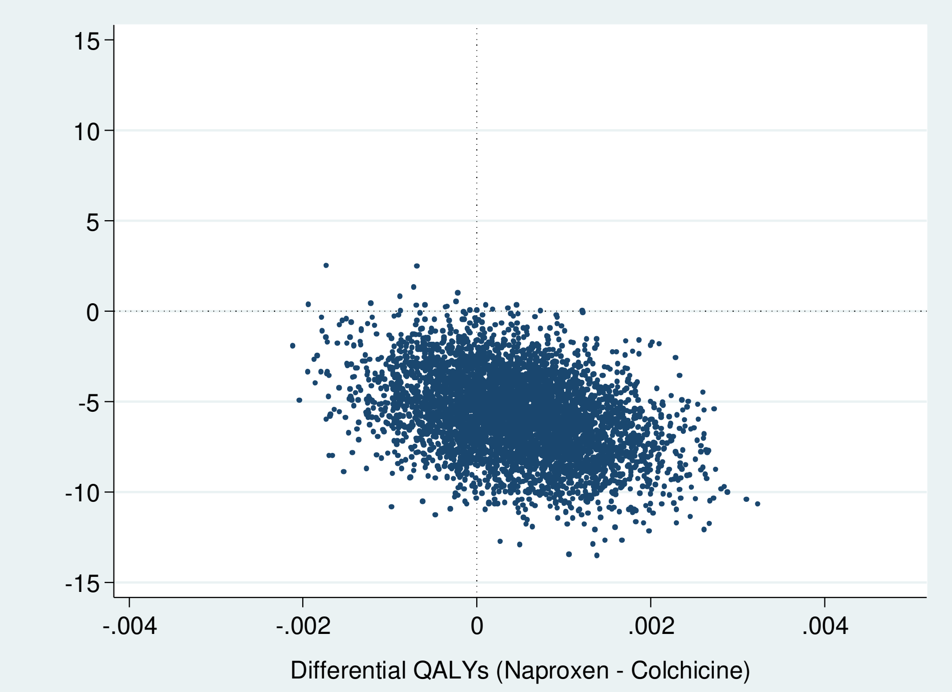

(B) Cost-effectiveness acceptability curve for Naproxen vs Colchicine

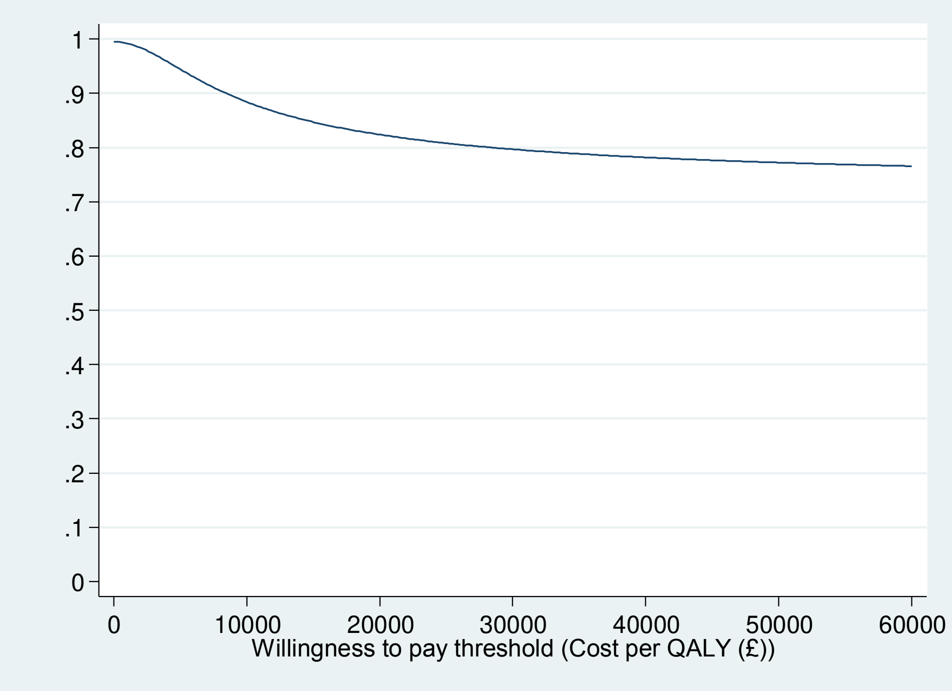

Supplement: Supplementary data [file annrheumdis-2019-216154supp002.pdf]
